# Co-occurrence of severe PTSD, somatic symptoms and dissociation in a large sample of childhood trauma inpatients: a network analysis

**DOI:** 10.1007/s00406-021-01342-z

**Published:** 2021-10-11

**Authors:** Leonhard Kratzer, Matthias Knefel, Alexander Haselgruber, Peter Heinz, Rebecca Schennach, Thanos Karatzias

**Affiliations:** 1 Department of Psychotraumatology, Clinic St. Irmingard, Prien am Chiemsee, Germany; 2 Faculty of Psychology, University of Vienna, Wächtergasse 1, 1010 Vienna, Austria; 3 Schoen Clinic Roseneck, Prien am Chiemsee, Germany; 4 Department of Psychiatry and Psychotherapy, Ludwig-Maximilians-University, Munich, Germany; 5 School of Health and Social Care, Edinburgh Napier University, Edinburgh, UK; 6 Rivers Centre for Traumatic Stress, NHS Lothian, Edinburgh, UK

**Keywords:** Comorbidity, Dissociative disorders, Pain, Posttraumatic stress disorder, Somatization

## Abstract

Co-occurrence of mental disorders including severe PTSD, somatic symptoms, and dissociation in the aftermath of trauma is common and sometimes associated with poor treatment outcomes. However, the interrelationships between these conditions at symptom level are not well understood. In the present study, we aimed to explore direct connections between PTSD, somatic symptoms, and dissociation to gain a deeper insight into the pathological processes underlying their comorbidity that can inform future treatment plans. In a sample of 655 adult inpatients with a diagnosis of severe PTSD following childhood abuse (85.6% female; mean age = 47.57), we assessed symptoms of PTSD, somatization, and dissociation. We analyzed the comorbidity structure using a partial correlation network with regularization. Mostly positive associations between symptoms characterized the network structure. Muscle or joint pain was among the most central symptoms. Physiological reactivation was central in the full network and together with concentrations problems acted as bridge between symptoms of PTSD and somatic symptoms. Headaches connected somatic symptoms with others and derealization connected dissociative symptoms with others in the network. Exposure to traumatic events has a severe and detrimental effect on mental and physical health and these consequences worsen each other trans-diagnostically on a symptom level. Strong connections between physiological reactivation and pain with other symptoms could inform treatment target prioritization. We recommend a dynamic, modular approach to treatment that should combine evidence-based interventions for PTSD and comorbid conditions which is informed by symptom prominence, readiness to address these symptoms and preference.

**Supplementary Information:**

The online version contains supplementary material available at 10.1007/s00406-021-01342-z.

## Introduction

Exposure to trauma is a major risk factor for poor mental and physical health [1] including Post-Traumatic Stress Disorder (PTSD), dissociation, depression, and anxiety disorders [2, 3] as well as physical consequences such as functional somatic syndromes, chronic pain disorders, and obesity [4, 5]. While the co-occurrence of these conditions is well documented in the literature (e.g. [6, 7]), their interrelationships at symptom level are not yet fully understood. The network model, which conceptualizes mental disorders as sets of interacting symptoms [8], where one symptom triggers another in a causal chain [9], provides a useful framework to understand these relationships among people with comorbid conditions. Patients who present with comorbidities present with challenges to mental health professionals with regard to which symptoms to target first. Network analysis can provide an insight into how these different symptoms relate to each other and which are the most central symptoms that may cause further psychopathology.

A typical presentation following child abuse includes co-occurrence of PTSD, somatization, and dissociation [10]. Co-occurrence of these conditions can sometimes lead to more treatment-resistant symptomatology [11, 12]. In the present study, we aimed to identify direct connections between PTSD, somatic symptoms, and dissociation to gain deeper insight into the pathological processes underlying their comorbidity.

Different pathways and mechanisms linking mental and physical health symptoms following traumatic experiences have been proposed and examined in the literature before [13]. Tsur [14] investigated the mediating role of Complex PTSD for the association of child abuse and chronic pain. While classic PTSD symptoms did not mediate this relationship, disturbances in self-organization (including affective dysregulation, a negative self-concept, and disturbances in relationships) mediated this association. In contrast, Morina and colleagues [15] found that the PTSD symptom cluster hyperarousal mediated the association of trauma exposure and somatic symptoms. Another approach was taken by Nijenhuis and colleagues [16], who proposed that somatic symptoms in trauma survivors can be seen as somatoform dissociation. As opposed to psychological dissociation, which includes the phenomena of depersonalization and derealization of the dissociative subtype of DSM-5 PTSD [17], somatoform dissociation phenomenologically involves the body and represents phenomena that are manifestations of a lack of integration of somatic experiences, reactions, and functions [16]. According to some authors, somatic dissociative symptoms can be seen as direct consequences of traumatic experiences [16], while others consider traumatic experiences only as major risk factor for somatoform dissociation [18]. Emphasizing yet a different aspect, Pace and Heim [19] argue that inflammatory and autoimmune changes in PTSD pathophysiology encourage later development of comorbid medical illnesses.

Although there is clear evidence for the common co-occurrence of PTSD, somatic symptoms, and dissociation, the direction of these associations and the underlying mechanisms, however, remain controversial and somatic symptoms are rather neglected in the classification of trauma-related disorders [20]. Possible pathways have been proposed and investigated including traumatic experiences as common cause of somatic symptoms, dissociation, and PTSD [1, 16], PTSD as mediator between trauma exposure and somatic symptoms [15], dissociation as mediator between childhood trauma and Complex PTSD [21], and dissociative or somatic symptoms as causally directly connected to symptoms of PTSD [22, 23]. Considering these ambiguous approaches and findings, in the current study, we hypothesized that the associations of symptoms are bidirectional. Utilizing a network analysis approach, we aimed to investigate direct associations of symptoms in a multivariate model, examining the unique relations between symptoms trans-diagnostically [24].

In research and practice, mental disorders have been conceptualized quite differently with crucial consequences for understanding comorbidity between disorders. A categorical understanding of mental disorders proposes that symptoms within the boundary of a disorder are manifestations of a shared common cause, the underlying, unobserved disease entity [25]. This approach is rooted in a traditional medical understanding of disease, where symptoms reflect an underlying pathology such as a malignant lung tumor that causes chest pain, bloody sputum, coughing, and breathlessness [26]. Adopting this model to psychopathology means that symptoms such as loss of interest, fatigue, and depressed mood are caused by depression. However, it is likely that there is no underlying root in the same sense when it comes to psychopathology [24], potentially limiting progress in research and treatment if this approach is operationalized. This might be particularly true when it comes to comorbidity. A symptom-level approach to the understanding of mental disorders might be more fruitful, even though most studies use categorical constructs on disorder or syndrome level to investigate the association of comorbid disorders [27]. The network approach on a symptom level does not assume the presence of an underlying disease entity.

While the proponents of both views agree with the notion that symptoms do not randomly co-occur, they disagree about the nature of the co-occurrence. The categorical perspective assumes a shared, underlying cause for both disorders whilst the symptom level-oriented network theory of mental disorders conceptualizes mental disorders as systems of interacting symptoms, recognizing that these interactions do not stop at the borders of categorical mental disorders [8]. Comorbidity can be understood as an intrinsic feature of mental disorders [27] and network analysis adds to the trans-diagnostic understanding of these psychopathological phenomena [28]. For example, researchers found that dysphoria-related symptoms connected PTSD and depression [29] and identified the feeling of being worthless and avoiding internal reminders of the stressor as central symptoms in a comorbidity network including PTSD, depression, and anxiety symptoms [30]. Looking beyond nosologically specified symptom sets, Kratzer and colleagues [31] identified sexual problems as connected to psychiatric symptoms in adult PTSD patients with childhood sexual abuse experiences. Difficulties engaging in sexual activities were linked to depressive and hyperarousal symptoms, whereas sexual preferences causing distress were linked to anger and dissociation [31]. However, a network perspective on psychiatric disorders does not necessarily preclude a classificatory approach to nosology in clinical practice. Diagnostic categories can provide important information for researchers and clinicians when it comes to semantic interoperability, treatment options, and outcome prediction. The network approach would only contradict a categorical classification system if disorders become reified [32].

In the current study, we hypothesized that the level of analysis plays a crucial role in understanding the comorbidity of disorders and applied network analysis as more appropriate and accurate approach of investigation than following a categorical understanding. In a recent study, Astill Wright and Colleagues [23] investigated the co-occurrence of PTSD and somatic symptoms from a network analytical perspective and found that sleeping difficulties may act as key bridge between PTSD and somatic symptoms. However, the approach taken in their study does not fully reflect the network perspective, as the analysis was on a domain level for PTSD. Thus, we used a network analytical framework to investigate the relationship between symptoms of somatization, dissociation, and PTSD on a symptom level. We aimed to investigate the structure of the emerging network, to identify the symptoms that are central in the network, and to detect especially important nodes in connecting the symptoms across the disorder boundaries to aid explaining mechanisms of comorbidity and co-occurrence. Overall, we aimed to provide evidence for the conception of psychiatric disorders and their comorbidity as networks of interacting symptoms.

## Method

The total sample comprised 655 adult inpatients (85.6% female; age: *M* = 47.57, *SD* = 10.20). All participants were diagnosed with ICD-10 PTSD following childhood abuse and were treated in the department of psychotraumatology of Clinic St. Irmingard, Germany. More than one-third (38.47%) were additionally diagnosed with a personality disorder (most frequently with Borderline Personality Disorder, 20.00% of the total sample), 37.40% were diagnosed with a somatoform disorder (most frequently with somatoform pain disorder, 23.81%), and 30.07% were diagnosed with a dissociative disorder. All diagnoses were clinical diagnoses given by attending psychologists and doctors relying on the structured clinical interview for DSM-IV personality disorders [33, 34] as well as the German version of the structured interview of disorders of extreme stress [35, 36]. At the time of admission, 65.2% received antidepressants, 29.3% received anxiolytics, 41.2% received antipsychotics, and 61.4% received analgesics. On average, patients reported *Md* = 3 inpatient and *Md* = 3 outpatient treatments prior to admission.

All psychometric tests were administered within 1 week after admission as part of the clinical routine assessment. Written informed consent was obtained from every patient. Due to the retrospective nature of our investigation, formal consent of the local ethics committee was not required.

Patients were administered the Childhood Trauma Questionnaire (CTQ; [37]) to retrospectively assess potentially traumatic childhood experiences. The CTQ consists of 25 items corresponding to the five subscales sexual abuse, physical abuse, emotional abuse, emotional neglect, and physical neglect. Patients indicate the severity of items like “Someone tried to make me do sexual things or watch sexual things.” on a five-point scale. The German version of the CTQ [38] has good psychometric properties, and exhibited satisfactory levels of internal consistency in the current study (*α* = 0.95).

The Impact of Event Scale-Revised (IES-R; [39]) was used to assess PTSD symptoms. The IES-R consists of 22 items like “I had dreams about it” that are answered on a 4-point scale and correspond to 3 subscales (intrusion, avoidance, hyperarousal). The IES-R was developed to assess posttraumatic symptoms and does not directly reflect the symptom set of a diagnostic manual. The psychometric properties of the German translation [40] were shown to be good and likewise exhibited satisfactory levels of internal consistency in the current study (*α* = 0.78).

The Hamburg Modules for the Assessment of Psychosocial Health (HEALTH-49; 41) questionnaire was used to assess somatic symptoms. It comprises nine subscales including somatoform complaints, depressiveness, and phobic anxiety. The somatoform complaints subscale was used in the present study to assess somatic symptoms. Each of the seven items of this subscale (e.g. “In the past two weeks, how much have you suffered from a feeling of heaviness in your arms and legs?”) is rated on a five-point scale. The resulting symptom score can be compared to norm data from inpatient psychotherapy patients. The psychometric properties of the German version are good [41] and the somatoform complaints subscale exhibited satisfactory levels of internal consistency in the current study (*α* = 0.83).

The Dissociative Experiences Scale—Taxon (DES-T; [42]) was developed to assess symptoms indicative of pathological dissociation. We included 1 item for depersonalization and 1 item for derealization that could be rated on a numerical 11-point scale to assess dissociative symptomatology according to the dissociative subtype of DSM-5 PTSD. The German version offers good psychometric properties [43] and the two items exhibited satisfactory levels of internal consistency in the current study (*α* = 0.71).

R version 4.0.2 [44] and the R packages qgraph version 1.6.5 [45], networktools version 1.2.3 [46], mgm version 1.2.10 [47], and bootnet version 1.4.3 [48] were used for data analysis.

The network approach to psychopathology allows visualizing the multivariate interdependencies of symptoms. In a symptom network, nodes represent symptoms and edges reflect pairwise relations between these symptoms. For our analysis, 22 PTSD symptoms, seven somatic symptoms, and two dissociative symptoms were included in the network estimation procedure. Using partial polychoric correlations, we investigated the connectivity of each symptom while controlling for all other associations in the network. To control for the possibility of false positive associations, we used the glasso regularization and a tuning parameter gamma set to 0.5 [49], thereby setting small edges, which are likely due to noise, exactly to zero and regularizing the network [50].

The Fruchtermann-Reingold algorithm [51] was used to place nodes with more and/or stronger connections more closely together. The maximum edge value was set to the strongest edge identified in the network (0.44) and the minimum edge value was set to 0.03 to enhance interpretability. We set positive edges to be printed in solid green lines and negative ones in dashed red lines. Stronger connections are indicated by more saturated and thicker edges. Importantly, the Fruchtermann-Reingold algorithm fosters readability but does not allow for a meaningful interpretation of the distances between nodes.

We used four parameters to describe the connectedness of each node in the estimated network: predictability, the centrality indices of node strength and expected influence, and bridge expected influence. Predictability is a characterization of symptom networks that gives an absolute measure of the controllability of each node. It is defined as the proportion of explained variance of a node by all other nodes. It thus quantifies how well a given node can be predicted by all other nodes it is connected to in the network [52]. We estimated the overall predictability of the nodes in the network as well as the predictability of each node.

Following recommendations from recent methodological work [53, 54], we used strength centrality to analyze the direct connections of nodes. Reflecting the sum of all absolute edge weights a node is directly connected to, strength centrality quantifies the connectivity of a node to all other nodes of the network. To take the potential importance of negative edges in symptom networks into account, we also estimated expected influence as additional centrality metric. In networks consisting of symptoms of different psychiatric disorders, it is also important to consider bridge centrality [55]. Bridge symptoms in a network are symptoms that work as a link between groups of disorder-specific symptoms and may, therefore, be helpful in explaining comorbidity. Hence, we also analyzed which symptoms are of importance in the comorbidity of PTSD, dissociation, and somatic symptoms. Bridge expected influence (1-step) was chosen as outcome parameter as recommended when negative edges are present. Bridge expected influence (1-step) is defined as the sum of the values of all edges between a node and all nodes from different communities. Finally, we calculated the mean of the absolute values of all edges that connect any two symptom communities (i.e., the three dimensions of PTSD, somatic symptoms, and dissociative symptoms) to investigate the average connectedness between any two symptom communities and to identify the PTSD dimension with the strongest connection to both the somatic and the dissociative symptoms communities. For this instance, we used Fisher’s *z*-transformation to average the edge weights.

To assess accuracy of the edge weight estimates, we conducted the routine implemented in the bootnet package [53], using nonparametric bootstrapping based on 2000 bootstrap samples to estimate 95% confidence intervals of all edge weights. To assess accuracy of the centrality estimates (strength, expected influence), we used the subsetting bootstrap function implemented in the bootnet package using 2000 samples with dropped cases. High correlations of the original centrality metric with the estimates from re-estimated networks indicate high stability. We then applied a correlation stability analysis. The correlation stability coefficient reflects the maximum number of dropped cases to retain a 95% probability of a correlation of at least *r* = 0.7 between the parameters of the original network and the parameters of the dropped cases networks and should not be below 0.25 [45].

## Results

### Descriptive statistics

Patients reported severe child abuse and neglect in the CTQ (*M* = 76.94; *SD* = 23.08) as well as severe PTSD symptoms in the IES-R (*M* = 82.82; *SD* = 13.45). Following the definition of Häuser et al. [56], participants experienced at least moderate-to-severe abuse and neglect: 81.8% emotional neglect, 80.3% emotional abuse, 71.9% physical neglect, 71.1% sexual abuse, and 61.1% physical abuse. Participants experienced at least moderate-to-severe abuse and neglect in several categories: 41.6% all five categories, 23.4% four categories, 14.6% three categories, 6.6% two categories, 7.5% one category, and 6.3% experienced child abuse and neglect that did not reach the moderate-to-severe cut off in any category. Compared to inpatient psychotherapy patients, 92.1% of the participants had a somatic symptom score (somatoform complaints subscale of the HEALTH-49) equal to or above the 90-percentile rank, hinting to the severe somatic symptomatology of the sample. Descriptive statistics of all assessed symptoms and types of abuse are reported in Table 1. The correlation matrix of all variables included in the network model is reported in Table S1.

**Table 1:** Means and standard deviations of relevant variables

Variable	*M*	*SD*	Item
INTR1	4.63	1.01	Any reminder brought back feelings about it
INTR2	4.36	1.07	Other things kept making me think about it
INTR3	4.30	1.20	I thought about it when I didn’t mean to
INTR4	4.20	1.33	Pictures about it popped into my mind
INTR5	3.73	1.66	I found myself acting or feeling like I was back at that time
INTR6	3.88	1.43	I had waves of strong feelings about it
INTR7	3.42	1.87	I had dreams about it
AVOID1	3.58	1.71	I avoided letting myself get upset when I thought about it or was reminded of it
AVOID2	1.98	2.04	I felt as if it hadn’t happened or wasn’t real
AVOID3	3.94	1.60	I stayed away from reminders of it
AVOID4	4.05	1.50	I tried not to think about it
AVOID5	2.51	1.97	I was aware that I still had a lot of feelings about it, but I didn’t deal with them
AVOID6	2.07	1.98	My feelings about it were kind of numb
AVOID7	3.35	1.96	I tried to remove it from my memory
AVOID8	3.98	1.64	I tried not to talk about it
HYP1	4.47	1.20	I had trouble staying asleep
HYP2	3.50	1.79	I felt irritable and angry
HYP3	4.23	1.34	I was jumpy and easily startled
HYP4	3.94	1.62	I had trouble falling asleep
HYP5	4.39	1.17	I had trouble concentrating
HYP6	4.28	1.31	Reminders of it caused me to have physical reactions, such as […]
HYP7	4.15	1.47	I felt watchful and on-guard
SOM1	2.63	1.43	Back pains
SOM2	2.29	1.41	Stomach pains or digestive problems
SOM3	2.50	1.26	Feeling of weakness in individual body parts
SOM4	2.28	1.41	Feeling of heaviness in arms and legs
SOM5	2.71	1.32	Pain in your muscles or joints
SOM6	2.38	1.37	Headaches or face pains
SOM7	2.09	1.40	Numbness or tingling in individual body parts
DISS1	26.70	30.55	… feeling as though they are standing next to themselves or watching themselves […]
DISS2	25.83	30.25	… feeling that other people, objects, and the world around them are not real
Emotional abuse	18.02	5.88	Verbal assaults on a child’s sense of worth or well-being or any humiliating or demeaning behavior directed toward a child by an adult or older person^a^
Physical abuse	12.63	6.24	Bodily assaults on a child by an adult or older person that posed a risk of or resulted in injury^a^
Sexual abuse	14.30	7.45	Sexual contact or conduct between a child younger than 18 years of age and an adult or older person^a^
Emotional neglect	19.44	5.38	The failure of caretakers to meet children’s basic emotional and psychological needs, including love, belonging, nurturance, and support^a^
Physical neglect	12.55	4.74	The failure of caretakers to provide for a child’s basic physical needs, including food, shelter, clothing, safety, and health care^a^

PTSD symptoms assessed with IES-R, somatic symptoms assessed with HEALTH-49, dissociation assessed with DES-T, child abuse and neglect assessed with CTQ

^a^[37]

### Network estimation

**Fig. 1:**
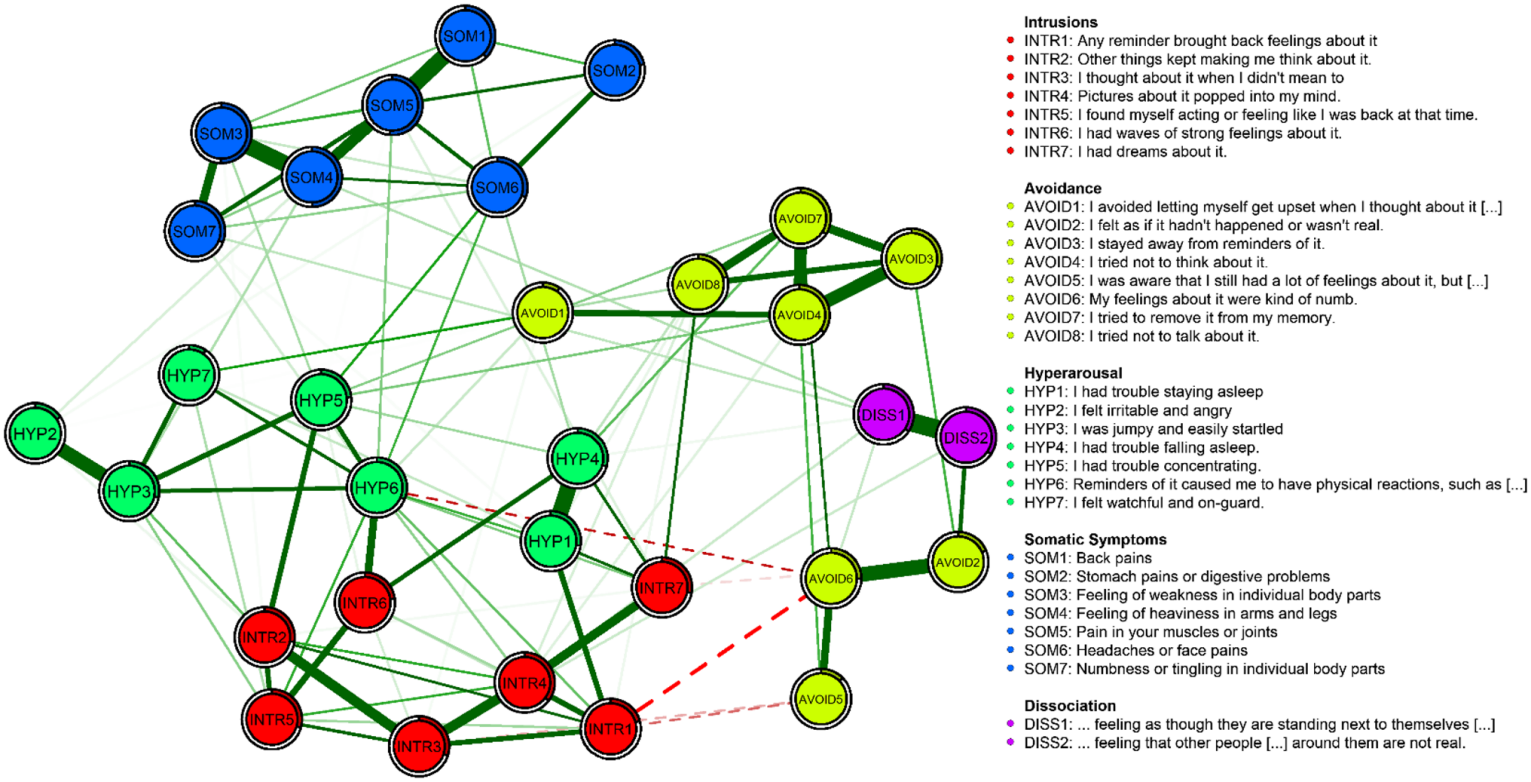
Network of symptoms of PTSD, somatization, and dissociation

Figure 1 depicts the symptom network. The majority of identified associations was positive and 173 (37.2% of 465) of the possible edges were estimated to be non-zero, indicating that, on average, a symptom is connected to more than one-third of all other symptoms in the network. The strongest association found in the network emerged between the two symptoms of dissociation. Other associations of particular strength were found between feeling of weakness (SOM3) and feeling of heaviness (SOM4) as well as between having troubles falling (HYP1) and staying asleep (HYP4). Hyperarousal symptoms and intrusion symptoms were strongly interconnected; avoidance symptoms were strongly connected to each other and showed negative connections with symptoms of intrusion and hyperarousal. Symptoms of PTSD showed manifold connections to somatic symptoms, while dissociative symptoms were weaker connected to both, PTSD and somatic symptoms. However, among the dissociative symptoms, derealization (DISS2) showed a relatively strong connection to the feeling that the traumatic event(s) had not happened (AVOID2).

### Network inference

**Fig. 2:**
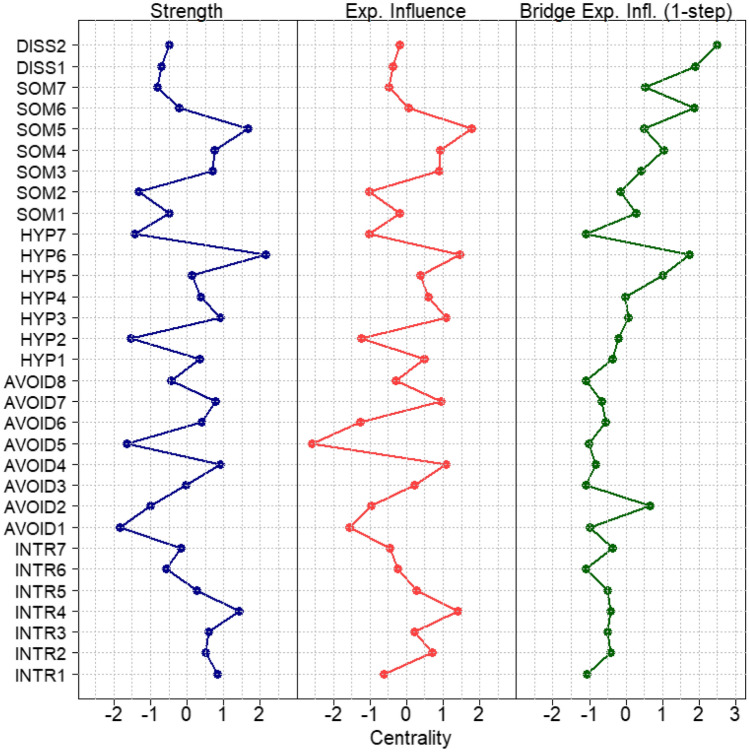
Centrality estimates

The standardized strength centrality estimates are shown in Fig. 2 (for a full list of exact parameters, please see Table S2). The mean predictability (illustrated by the percentage of shaded area in the pie around the nodes in Fig. 1) of the full network was 0.44, indicating that on average, 44% of the variation of each symptom could be explained by its neighboring symptoms. The symptom that could best be predicted by neighboring symptoms, i.e. the node with the highest predictability, was muscle or joint pain (SOM5) and the symptom with the lowest predictability was avoiding getting upset when reminded of the trauma (AVOID1). The variation of this latter symptom was most independent from its neighbors. We found that physiological reactivation (HYP6) had the highest strength centrality estimate and muscle or joint pain (SOM5) had the highest expected influence estimate. i.e., these symptoms showed the strongest average connections to other symptoms. The correlation between the standard deviation of the nodes with strength and expected influence was low (|*r*|< 0.18). The nodes with the highest bridge expected influence were physiological reactivation (HYP6) and concentration problems (HYP5) from the PTSD symptoms, headaches (SOM6) from the somatic symptoms, and derealization (DISS2) from the dissociative symptoms. This means that these symptoms are particularly relevant in connecting the three communities. The strongest average connection between any of the three PTSD dimensions (intrusions, avoidance, and hyperarousal) and the somatic symptoms was found for hyperarousal, the strongest connection between the PTSD dimensions and dissociation was found for avoidance (Table S3).

### Accuracy and stability

The edge weight bootstrap analysis (shown in Fig. S1) reflects an accurately estimated network with strong edges being substantially larger than zero. The subset bootstrapping analysis showed sufficient stability of the strength and expected influence centrality as well as the bridge expected influence centrality estimate (shown in Fig. S2). The correlation stability (CS) coefficients were found to be CS = 0.67 for strength and expected influence and CS = 0.52 for bridge expected influence. The centrality difference test showed that the nodes with the highest centrality differed significantly from the centrality of most other nodes (shown in Fig. S3).

## Discussion

To the best of our knowledge, our study represents the first symptom-level network analysis of PTSD, somatic symptoms, and dissociation in a sample of adult inpatients with severe PTSD. The network structure was characterized by mostly positive associations between symptoms; strongly connected symptoms of dissociation, strongly connected somatic symptoms, and relatively strong connections of symptoms within the three dimensions of PTSD (intrusion, avoidance, hyperarousal). The symptoms with the highest centrality were physiological reactivation and muscle or joint pain. The symptoms with the strongest connections across the disorder boundaries were physiological reactivation and concentration problems from the PTSD symptoms, headaches from the somatic symptoms, and derealization from the dissociative symptoms.

Consistent with previous research, we found manifold connections between the three symptom groups we investigated. Importantly, the symptoms tended to show stronger connections within their respective group, supporting the clinically relevant categorical approach of diagnosing mental disorders. Co-occurrence of symptoms is the basis for current classification systems. This categorical approach clusters symptoms to syndromes (disorders) when they co-occur frequently and the resulting system provides a common language and important information on treatment options and outcome prediction for researchers and clinicians [57]. Consistent with this approach, symptoms within one diagnostic category were stronger connected to each other in our study. However, when clinically useful categories become reified, which has been often the case [32], scientific progress may be impeded [58]. In the following sections, we will thus discuss our results from a symptom-level perspective.

Interestingly and in contrast to previous research, the PTSD hyperarousal symptoms in general and specifically concentration problems and physiological reactivation showed the strongest connections to somatic symptoms. Astill Wright and colleagues [23] found that symptoms of re-experiencing showed the strongest connection while alterations in arousal and reactivity showed the weakest connection to somatic symptoms in their sample. Several aspects may explain these diverging findings. First, our study included only inpatients requiring trauma-specific treatment. Astill Wright and colleagues [23] excluded potential participants from their study if they had recently required inpatient treatment or had frequent contact with a crisis-related intensive home treatment. This resulted in an almost complementary selection of participants. Second, the sample in the present paper represents a group of adults with a history of child abuse as well as long lasting and severe experiences of all types of child abuse and neglect, while the previous study included individuals with any type of traumatic experience. Even though all participants in our and the previous study fulfilled the criteria for PTSD, our sample can be seen as a group of people who might be characterized as more severe PTSD cases. Third, we investigated the relationship between PTSD and somatization on a symptom level, which allows for a more detailed analysis of symptom dynamics.

Symptoms of PTSD and somatization may most likely mutually maintain each other [59, 60]. Different dimensions of PTSD have been associated with somatic symptoms. Ulirsch and colleagues [61] found that the avoidance symptom cluster was predictive for the number of regions with new pain symptoms in a sample of sexual assault survivors. Ravn et al. [62] identified the hyperarousal symptom cluster as a driving force behind the association of PTSD and pain. While the current study supports the finding that hyperarousal symptoms play a crucial role in the connection of PTSD and somatic symptoms, it also highlights the complexity of symptom interaction that cannot be reduced to sum-scores without loss of relevant information. For example, although the hyperarousal symptoms show the strongest connection to somatic symptoms, single symptoms of other clusters are associated with somatic symptoms as well, such as the connection between having nightmares and pain in muscles or joints.

In the current sample with severe experiences of child abuse, we argue that the importance of hyperarousal symptoms needs to be viewed from a bio-psycho-social perspective. Heightened threat processing at multiple levels, including social information processing biases, altered emotional learning, elevated emotional reactivity, and emotion regulation difficulties as consequence of child abuse [63], may lead to low precision in relaying interoceptive information in the brain and thus other factors such as cognitions come to dominate the perception of the health status of the body [64]. Negative posttraumatic cognitions about the self, such as feelings of worthlessness, are in turn often at the core of posttraumatic stress symptomatology [65] and may thus enhance dysfunctional perceptions of the body. Hyperarousal in PTSD alters the stress response of the body via the Hypothalamic–Pituitary–Adrenal (HPA) axis [66]. Dysfunction in the HPA axis has in turn been associated with somatic syndromes (e.g. irritable bowel syndrome [67]). Taken the cross-sectional nature of our study into account, we cannot speculate about the causal direction of the associations, but in accordance with previous research, we hypothesize that the interrelation of PTSD and somatic symptoms is based on a mutual interaction on symptom level that has neurobiological, psychological, and social determinants.

Dissociative symptoms are a frequently observed phenomenon following traumatic stress [68–70], which has led to the inclusion of a dissociative subtype of PTSD in DSM-5 characterized by depersonalization and derealization [17]. In our study, we found that these two symptoms of dissociation are closely related, replicating previous results [18, 22, 71]. Derealization was the symptom with higher centrality in all measures; however, the difference compared to depersonalization was not significant. Dissociative symptoms showed the strongest average connection to symptoms of avoidance, and derealization was particularly strongly connected to the avoidance symptom of having the feeling that the traumatic event did not happen or was not real. This is indeed supported by psychological theories of PTSD which imply a dissociation of emotional, perceptual, and episodic memory for the traumatic event(s) as a core feature of PTSD [72, 73]. This fragmentation may be a starting point for unexplained somatic symptoms, closing the cycle of trauma, PTSD, dissociation, and somatic symptoms. Future research should also include measures of ICD-11 Complex PTSD, particularly in samples with severe experiences of childhood abuse. In a network analytical comorbidity study including Complex PTSD symptoms, dissociative symptoms were among the most central [71].

The network model of mental disorders emphasizes the importance of symptom interaction and has consequences for treatment. The centrality hypothesis recognizes central symptoms as primary treatment targets but has received conflicting empirical support so far [74]. In this line, we argue that picking out single symptoms as treatment targets does not follow the core assumption of the network approach, which is that mental disorders arise from the direct interactions between symptoms [8]. Consequently, focusing on the associations of symptoms might be a more successful strategy of intervention. In our study, we found that that physiological reactivation had the highest centrality estimate. It showed particularly strong connections to other hyperarousal symptoms, the intrusion symptom having strong waves of feelings about the trauma, and the somatic symptom headaches or face pains. Interventions targeting these connections could help patients break up the link between the respective symptoms. Learning to tolerate and accept physical aspects of emotions such that physical reactions that accompany strong feelings are not experienced as disabling [75] could, therefore, disconnect these symptoms. Similarly, the connection between headaches and face pains and physiological reactivation could be reduced by pharmacological [76] and psychological therapy [77]. Finally, the connections of hyperarousal symptoms with each other might be reduced by state-of-the-art trauma-therapy approaches such as prolonged exposure [78]. Imaginal exposure (repeated recounting of the most disturbing traumatic memory) and listening to audio recordings of the imaginal recounting is purported to reduce physiological reactivation and in vivo exposure (approaching trauma-related situations) to reduce hypervigilance. Modular approaches [79] provide a useful framework for the treatment of severe traumatization and advocate that individual symptoms should be targeted in therapy using a formulation-based approach guided by symptom severity, preference to target a symptom and readiness to change this symptom. Following this approach, PTSD symptoms, dissociation, or somatization can be prioritized for treatment using the aforementioned criteria. Yet, a noteworthy limitation regarding this reasoning is the extensive prior treatment experience in the sample with 574 patients (87.6%) having received at least one prior psychiatric inpatient treatment (median = 3, max = 49) and 631 patients (96.3%) having received at least one outpatient psychotherapeutic treatment (median = 2, max = 8). It remains unclear whether modular approaches as suggested were part of these treatments.

### Limitations

The present study has a number of limitations. Even though the inclusion of participants with PTSD was based on clinical interviews, the data used for the analysis in the present study are based on patients’ self-reports. The assessment of dissociation included only two items, reflecting the DSM-5 dissociative subtype of PTSD formulation, whereas dissociative phenomena may manifest in different ways as well. The cross-sectional nature of the presented analysis does not allow for causal inference and interpretations of the direction of associations should be done carefully. Finally, our results may generalize only to other treatment samples and similarities and differences to other samples still need to be investigated.

## Conclusions

In conclusion, the sequelae of traumatic stress do not end at the boarders defined by classification manuals. Trauma has a severe and detrimental effect on mental and physical health and these consequences worsen each other trans-diagnostically on a symptom level. Dissociative and somatic symptoms have been shown to negatively affect treatment outcomes in the treatment of PTSD [12]. Interventions aiming at the improvement of trauma sequelae should thus address the individual symptom profile of each patient. A dynamic, modular approach to treatment [79] should include evidence-based interventions for PTSD [80] and comorbid symptoms [81]. An investigation of strong connections between symptoms in individual trans-diagnostic symptom networks could inform treatment target prioritization and sequencing of symptom targeting.

## Supplementary Information

Below is the link to the electronic supplementary material.

Supplementary file1 (DOCX 219 KB)
