# Supplementary material for: Co-occurrence of severe PTSD, somatic symptoms and dissociation in a large sample of childhood trauma inpatients: a network analysis
Source: Eur Arch Psychiatry Clin Neurosci. 2021 Oct 11;272(5):897–908. doi: 10.1007/s00406-021-01342-z (PMC9279203; doi:10.1007/s00406-021-01342-z)
Supplement: Supplementary file 1 — Supplementary file1 (DOCX 219 KB) [file 406_2021_1342_MOESM1_ESM.docx]

**Supplementary Materials**

**for**

**Co-occurrence of severe PTSD, Somatic Symptoms and Dissociation in a large sample of childhood trauma inpatients: A Network Analysis**

Kratzer, Leonhard^1^*; Knefel, Matthias^2^*; Haselgruber, Alexander^2^; Heinz, Peter^1^; Schennach, Rebecca^3^; Karatzias, Thanos^4^;

^1^Department of Psychotraumatology, Clinic St. Irmingard, Prien am Chiemsee, Germany

^2^Faculty of Psychology*,* University of Vienna, Austria

^3^Schoen Clinic Roseneck, Prien am Chiemsee, Germany

Department of Psychiatry and Psychotherapy, Ludwig-Maximilians-University, Munich, Germany

^4^School of Health & Social Care, Edinburgh Napier University, Edinburgh, UK

Rivers Centre for Traumatic Stress, NHS Lothian, Edinburgh, UK

*shared first authorship.

Correspondence concerning this article should be addressed to Matthias Knefel, Faculty of Psychology*,* University of Vienna, Wächtergasse 1, 1010 Vienna, Austria, phone: +43 1 4277 47247, e-mail: matthias.knefel@univie.ac.at

Table S1. Correlation matrix of all variables in the network model.

|  | 1 | 2 | 3 | 4 | 5 | 6 | 7 | 8 | 9 | 10 | 11 | 12 | 13 | 14 | 15 | 16 | 17 | 18 | 19 | 20 | 21 | 22 | 23 | 24 | 25 | 26 | 27 | 28 | 29 | 30 | 31 |
| --- | --- | --- | --- | --- | --- | --- | --- | --- | --- | --- | --- | --- | --- | --- | --- | --- | --- | --- | --- | --- | --- | --- | --- | --- | --- | --- | --- | --- | --- | --- | --- |
| 1. INTR1 | 1,00 |  |  |  |  |  |  |  |  |  |  |  |  |  |  |  |  |  |  |  |  |  |  |  |  |  |  |  |  |  |  |
| 2. INTR2 | 0,45 | 1,00 |  |  |  |  |  |  |  |  |  |  |  |  |  |  |  |  |  |  |  |  |  |  |  |  |  |  |  |  |  |
| 3. INTR3 | 0,49 | 0,56 | 1,00 |  |  |  |  |  |  |  |  |  |  |  |  |  |  |  |  |  |  |  |  |  |  |  |  |  |  |  |  |
| 4. INTR4 | 0,51 | 0,49 | 0,57 | 1,00 |  |  |  |  |  |  |  |  |  |  |  |  |  |  |  |  |  |  |  |  |  |  |  |  |  |  |  |
| 5. INTR5 | 0,40 | 0,47 | 0,46 | 0,46 | 1,00 |  |  |  |  |  |  |  |  |  |  |  |  |  |  |  |  |  |  |  |  |  |  |  |  |  |  |
| 6. INTR6 | 0,36 | 0,32 | 0,35 | 0,39 | 0,45 | 1,00 |  |  |  |  |  |  |  |  |  |  |  |  |  |  |  |  |  |  |  |  |  |  |  |  |  |
| 7. INTR7 | 0,34 | 0,34 | 0,30 | 0,50 | 0,31 | 0,26 | 1,00 |  |  |  |  |  |  |  |  |  |  |  |  |  |  |  |  |  |  |  |  |  |  |  |  |
| 8. AVOID1 | 0,19 | 0,16 | 0,19 | 0,19 | 0,14 | 0,15 | -0,03 | 1,00 |  |  |  |  |  |  |  |  |  |  |  |  |  |  |  |  |  |  |  |  |  |  |  |
| 9. AVOID2 | -0,15 | -0,01 | -0,05 | -0,10 | 0,04 | -0,02 | -0,14 | 0,02 | 1,00 |  |  |  |  |  |  |  |  |  |  |  |  |  |  |  |  |  |  |  |  |  |  |
| 10. AVOID3 | 0,18 | 0,12 | 0,17 | 0,25 | 0,21 | 0,14 | 0,09 | 0,14 | 0,21 | 1,00 |  |  |  |  |  |  |  |  |  |  |  |  |  |  |  |  |  |  |  |  |  |
| 11. AVOID4 | 0,22 | 0,19 | 0,22 | 0,28 | 0,15 | 0,20 | 0,11 | 0,32 | 0,01 | 0,58 | 1,00 |  |  |  |  |  |  |  |  |  |  |  |  |  |  |  |  |  |  |  |  |
| 12. AVOID5 | -0,25 | -0,13 | -0,21 | -0,19 | -0,18 | -0,06 | -0,15 | 0,07 | 0,13 | 0,11 | 0,17 | 1,00 |  |  |  |  |  |  |  |  |  |  |  |  |  |  |  |  |  |  |  |
| 13. AVOID6 | -0,29 | -0,15 | -0,14 | -0,12 | -0,14 | -0,12 | -0,11 | 0,00 | 0,47 | 0,08 | 0,02 | 0,35 | 1,00 |  |  |  |  |  |  |  |  |  |  |  |  |  |  |  |  |  |  |
| 14. AVOID7 | 0,11 | 0,11 | 0,10 | 0,26 | 0,21 | 0,14 | 0,19 | 0,17 | 0,17 | 0,55 | 0,59 | 0,10 | 0,20 | 1,00 |  |  |  |  |  |  |  |  |  |  |  |  |  |  |  |  |  |
| 15. AVOID8 | 0,25 | 0,12 | 0,04 | 0,29 | 0,22 | 0,13 | 0,31 | 0,07 | 0,01 | 0,43 | 0,35 | 0,04 | 0,06 | 0,47 | 1,00 |  |  |  |  |  |  |  |  |  |  |  |  |  |  |  |  |
| 16. HYP1 | 0,42 | 0,30 | 0,17 | 0,37 | 0,28 | 0,34 | 0,40 | 0,04 | -0,05 | 0,17 | 0,14 | -0,11 | -0,18 | 0,16 | 0,28 | 1,00 |  |  |  |  |  |  |  |  |  |  |  |  |  |  |  |
| 17. HYP2 | 0,16 | 0,25 | 0,17 | 0,21 | 0,15 | 0,09 | 0,08 | 0,16 | -0,03 | 0,06 | 0,04 | -0,06 | -0,13 | 0,04 | 0,03 | 0,13 | 1,00 |  |  |  |  |  |  |  |  |  |  |  |  |  |  |
| 18. HYP3 | 0,23 | 0,38 | 0,23 | 0,35 | 0,36 | 0,29 | 0,29 | 0,18 | -0,01 | 0,12 | 0,20 | -0,13 | -0,15 | 0,17 | 0,15 | 0,28 | 0,50 | 1,00 |  |  |  |  |  |  |  |  |  |  |  |  |  |
| 19. HYP4 | 0,21 | 0,30 | 0,23 | 0,31 | 0,30 | 0,39 | 0,39 | 0,01 | 0,03 | 0,22 | 0,11 | -0,08 | 0,00 | 0,29 | 0,29 | 0,60 | 0,17 | 0,28 | 1,00 |  |  |  |  |  |  |  |  |  |  |  |  |
| 20. HYP5 | 0,25 | 0,44 | 0,27 | 0,36 | 0,34 | 0,33 | 0,27 | 0,15 | -0,08 | 0,23 | 0,29 | -0,13 | -0,09 | 0,29 | 0,20 | 0,31 | 0,24 | 0,43 | 0,33 | 1,00 |  |  |  |  |  |  |  |  |  |  |  |
| 21. HYP6 | 0,42 | 0,31 | 0,34 | 0,44 | 0,44 | 0,49 | 0,38 | 0,22 | -0,12 | 0,13 | 0,22 | -0,14 | -0,25 | 0,17 | 0,26 | 0,41 | 0,22 | 0,46 | 0,32 | 0,46 | 1,00 |  |  |  |  |  |  |  |  |  |  |
| 22. HYP7 | 0,22 | 0,14 | 0,18 | 0,23 | 0,27 | 0,21 | 0,17 | 0,23 | -0,01 | 0,17 | 0,20 | 0,02 | -0,01 | 0,09 | 0,22 | 0,25 | 0,12 | 0,34 | 0,10 | 0,22 | 0,35 | 1,00 |  |  |  |  |  |  |  |  |  |
| 23. SOM1 | 0,16 | 0,18 | 0,12 | 0,21 | 0,19 | 0,17 | 0,24 | 0,02 | -0,05 | 0,07 | 0,08 | 0,02 | 0,05 | 0,19 | 0,15 | 0,24 | 0,16 | 0,27 | 0,20 | 0,23 | 0,32 | 0,03 | 1,00 |  |  |  |  |  |  |  |  |
| 24. SOM2 | -0,01 | 0,09 | 0,09 | 0,12 | 0,10 | 0,10 | 0,10 | 0,13 | 0,02 | 0,09 | 0,17 | 0,08 | 0,02 | 0,14 | 0,13 | 0,18 | 0,19 | 0,22 | 0,16 | 0,17 | 0,25 | 0,03 | 0,36 | 1,00 |  |  |  |  |  |  |  |
| 25. SOM3 | 0,15 | 0,28 | 0,19 | 0,23 | 0,25 | 0,16 | 0,16 | 0,04 | -0,02 | 0,02 | 0,04 | 0,00 | -0,09 | 0,08 | 0,06 | 0,12 | 0,18 | 0,31 | 0,18 | 0,34 | 0,34 | 0,11 | 0,45 | 0,36 | 1,00 |  |  |  |  |  |  |
| 26. SOM4 | 0,15 | 0,27 | 0,18 | 0,27 | 0,24 | 0,20 | 0,25 | 0,09 | -0,04 | 0,02 | 0,02 | -0,02 | -0,05 | 0,04 | 0,10 | 0,21 | 0,25 | 0,33 | 0,17 | 0,27 | 0,33 | 0,13 | 0,46 | 0,35 | 0,69 | 1,00 |  |  |  |  |  |
| 27. SOM5 | 0,16 | 0,19 | 0,16 | 0,27 | 0,17 | 0,21 | 0,28 | 0,14 | -0,07 | 0,02 | 0,10 | 0,05 | -0,05 | 0,10 | 0,11 | 0,24 | 0,16 | 0,27 | 0,18 | 0,34 | 0,39 | 0,13 | 0,65 | 0,42 | 0,58 | 0,65 | 1,00 |  |  |  |  |
| 28. SOM6 | 0,20 | 0,21 | 0,19 | 0,17 | 0,14 | 0,16 | 0,22 | 0,12 | -0,02 | 0,05 | 0,11 | 0,01 | 0,00 | 0,10 | 0,09 | 0,25 | 0,20 | 0,25 | 0,28 | 0,35 | 0,37 | 0,13 | 0,41 | 0,39 | 0,43 | 0,46 | 0,51 | 1,00 |  |  |  |
| 29. SOM7 | 0,10 | 0,18 | 0,21 | 0,22 | 0,22 | 0,16 | 0,19 | 0,10 | 0,01 | 0,08 | 0,15 | -0,04 | -0,05 | 0,09 | 0,07 | 0,24 | 0,13 | 0,20 | 0,16 | 0,23 | 0,31 | 0,10 | 0,37 | 0,30 | 0,53 | 0,47 | 0,50 | 0,37 | 1,00 |  |  |
| 30. DISS1 | 0,17 | 0,15 | 0,19 | 0,22 | 0,18 | 0,13 | 0,13 | 0,09 | 0,19 | 0,07 | 0,05 | -0,04 | 0,15 | 0,14 | 0,14 | 0,14 | 0,02 | 0,09 | 0,19 | 0,12 | 0,19 | 0,04 | 0,18 | 0,14 | 0,20 | 0,23 | 0,15 | 0,19 | 0,13 | 1,00 |  |
| 31. DISS2 | 0,04 | 0,19 | 0,21 | 0,12 | 0,19 | 0,11 | 0,11 | 0,06 | 0,26 | 0,08 | 0,02 | -0,02 | 0,15 | 0,16 | 0,11 | 0,07 | 0,07 | 0,12 | 0,11 | 0,16 | 0,16 | 0,06 | 0,11 | 0,13 | 0,19 | 0,20 | 0,13 | 0,12 | 0,20 | 0,55 | 1,00 |

Table S2. Raw scores of standardized centrality estimates.

|  | Strength | EI | Bridge EI (1-step) | Predictability |
| --- | --- | --- | --- | --- |
| INTR1 | 0.82 | -0.61 | -1.06 | 0.21 |
| INTR2 | 0.50 | 0.70 | -0.43 | 0.28 |
| INTR3 | 0.59 | 0.23 | -0.50 | 0.30 |
| INTR4 | 1.41 | 1.41 | -0.43 | 0.35 |
| INTR5 | 0.26 | 0.28 | -0.52 | 0.30 |
| INTR6 | -0.56 | -0.25 | -1.08 | 0.23 |
| INTR7 | -0.17 | -0.45 | -0.37 | 0.28 |
| AVOID1 | -1.83 | -1.55 | -1.00 | 0.09 |
| AVOID2 | -0.99 | -0.97 | 0.66 | 0.19 |
| AVOID3 | -0.03 | 0.22 | -1.08 | 0.30 |
| AVOID4 | 0.91 | 1.07 | -0.83 | 0.35 |
| AVOID5 | -1.66 | -2.60 | -1.00 | 0.13 |
| AVOID6 | 0.40 | -1.26 | -0.55 | 0.25 |
| AVOID7 | 0.77 | 0.94 | -0.67 | 0.34 |
| AVOID8 | -0.43 | -0.29 | -1.08 | 0.21 |
| HYP1 | 0.34 | 0.48 | -0.38 | 0.27 |
| HYP2 | -1.55 | -1.24 | -0.19 | 0.16 |
| HYP3 | 0.92 | 1.08 | 0.07 | 0.28 |
| HYP4 | 0.38 | 0.60 | -0.03 | 0.29 |
| HYP5 | 0.14 | 0.38 | 1.01 | 0.24 |
| HYP6 | 2.14 | 1.46 | 1.73 | 0.31 |
| HYP7 | -1.43 | -1.03 | -1.08 | 0.09 |
| SOM1 | -0.48 | -0.18 | 0.29 | 0.38 |
| SOM2 | -1.34 | -1.02 | -0.16 | 0.22 |
| SOM3 | 0.70 | 0.89 | 0.40 | 0.50 |
| SOM4 | 0.75 | 0.93 | 1.03 | 0.50 |
| SOM5 | 1.68 | 1.77 | 0.49 | 0.52 |
| SOM6 | -0.22 | 0.06 | 1.86 | 0.30 |
| SOM7 | -0.81 | -0.48 | 0.52 | 0.31 |
| DISS1 | -0.71 | -0.38 | 1.90 | 0.34 |
| DISS2 | -0.50 | -0.19 | 2.48 | 0.34 |

|  | 1 | 2 | 3 | 4 | 5 |
| --- | --- | --- | --- | --- | --- |
| 1 Intrusion | - |  |  |  |  |
| 2 Avoidance | .013 | - |  |  |  |
| 3 Hyperarousal | .032 | .012 | - |  |  |
| 4 Somatic symptoms | .003 | .001 | .014 | - |  |
| 5 Dissociation | .010 | .013 | .003 | .011 | - |

Table S3. Average connections between communities.

Figure S1. Bootstrap 95% confidence intervals for the edge weights in the symptom network. The labels of the edges/symptoms on the y-axis have been removed to avoid cluttering. The red line shows the sample edge weight values and the grey areas show the respective bootstrapped confidence intervals.


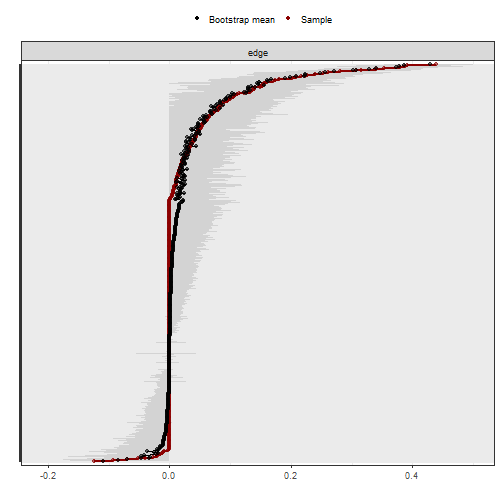

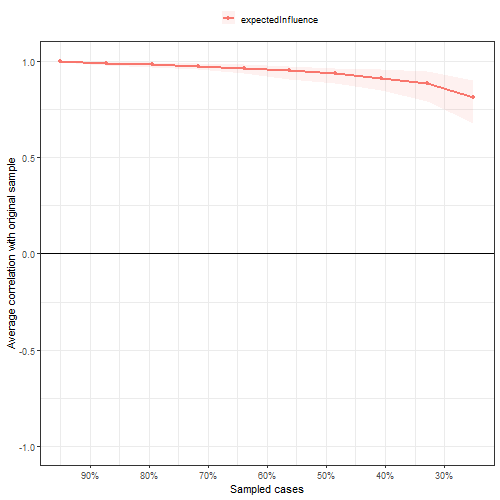

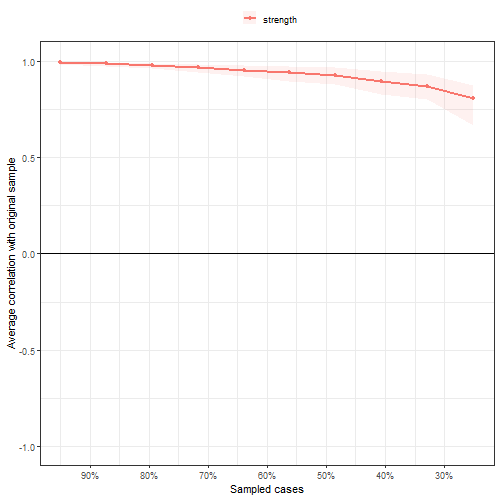

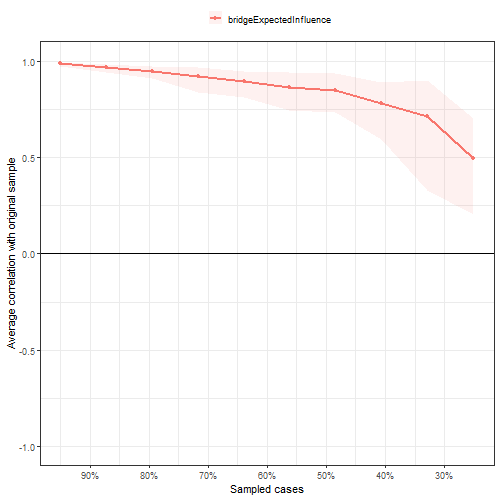


Figure S2. Top: Results of the subsetting bootstrap analysis reflect high correlations of the node strength centrality (left) and expected influence (right) for the original network and the indices of networks constructed with subsets of the original sample. Bottom: Results of the subsetting bootstrap analysis reflect high correlations of the bridge expected influence centrality for the original network and the indices of networks constructed with subsets of the original sample.


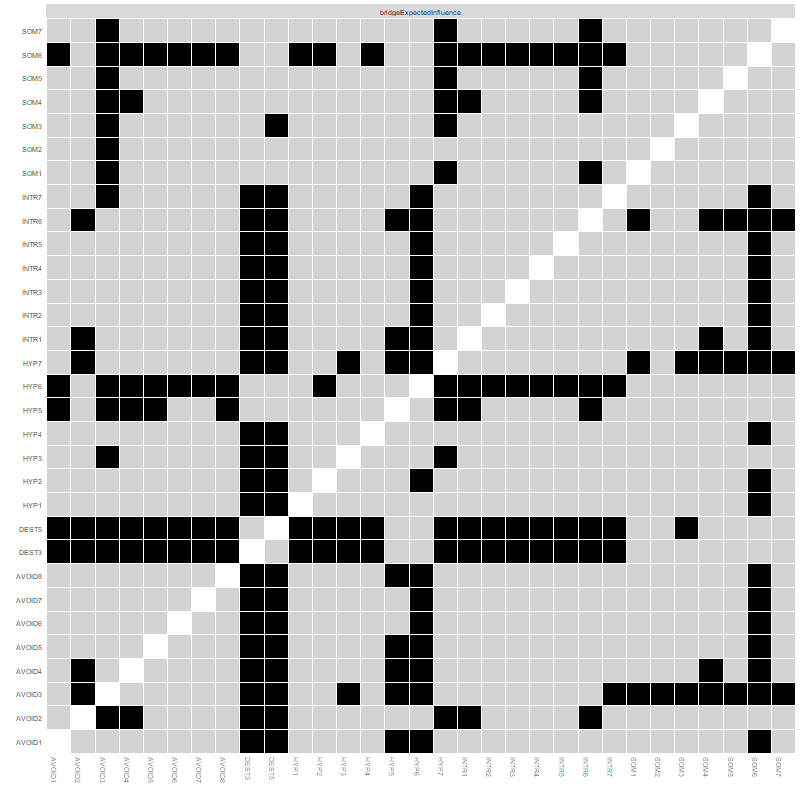

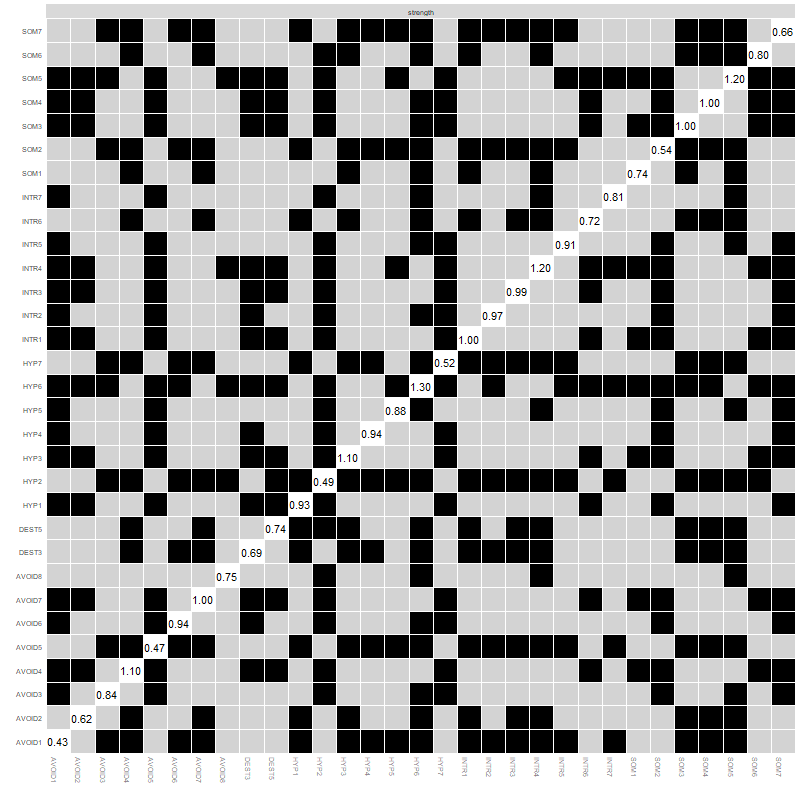

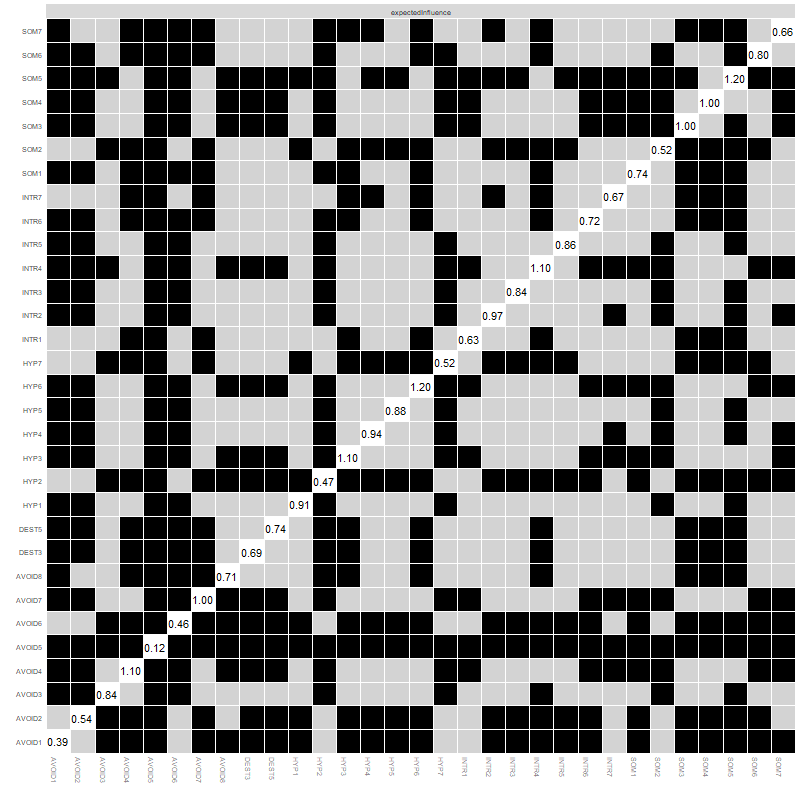


Figure S3. Centrality difference test. Standardized centrality values are shown in the diagonal, black boxes represent significant differences in centrality estimates (top left: strength; top right: expected influence; bottom: bridge expected influence). The test does not correct for multiple testing.
